# Expression and Regulation of PIWIL-Proteins and PIWI-Interacting RNAs in Rheumatoid Arthritis

**DOI:** 10.1371/journal.pone.0166920

**Published:** 2016-11-28

**Authors:** Lenka Pleštilová, Michel Neidhart, Giancarlo Russo, Mojca Frank-Bertoncelj, Caroline Ospelt, Adrian Ciurea, Christoph Kolling, Renate E. Gay, Beat A. Michel, Jiří Vencovský, Steffen Gay, Astrid Jüngel

**Affiliations:** 1 Center of Experimental Rheumatology, University Hospital Zürich, Zürich, Switzerland; 2 Functional Genomic Centre Zürich, Zürich, Switzerland; 3 Department of Rheumatology, University Hospital Zürich, Zürich, Switzerland; 4 Schulthess Clinic, Zürich, Switzerland; 5 Institute of Rheumatology and Clinic of Rheumatology, 1st Faculty of Medicine, Charles University in Prague, Prague, Czech Republic; Universite de Nantes, FRANCE

## Abstract

**Objective:**

The PIWIL (P-element induced wimpy testis like protein) subfamily of argonaute proteins is essential for Piwi-interacting RNA (piRNA) biogenesis and their function to silence transposons during germ-line development. Here we explored their presence and regulation in rheumatoid arthritis (RA).

**Methods:**

The expression of PIWIL genes in RA and osteoarthritis (OA) synovial tissues and synovial fibroblasts (SF) was analysed by Real-time PCR, immunofluorescence and Western blot. The expression of piRNAs was quantified by next generation small RNA sequencing (NGS). The regulation of PIWI/piRNAs, proliferation and methylation of LINE-1 after silencing of PIWIL genes were studied.

**Results:**

PIWIL2 and 4 mRNA were similarly expressed in synovial tissues and SF from RA and OA patients. However, on the protein level only PIWIL4 was strongly expressed in SF. Using NGS up to 300 piRNAs were identified in all SF without significant differences in expression levels between RA and OASF. Of interest, the analysis of the co-expression of the detected piRNAs revealed a less tightly regulated pattern of piRNA-823, -4153 and -16659 expression in RASF. In RASF and OASF, stimulation with TNFα+IL1β/TLR-ligands further significantly increased the expression levels of PIWIL2 and 4 mRNA and piRNA-16659 was significantly (4-fold) induced upon Poly(I:C) stimulation. Silencing of PIWIL2/4 neither affect LINE-1 methylation/expression nor proliferation of RASF.

**Conclusion:**

We detected a new class of small regulatory RNAs (piRNAs) and their specific binding partners (PIWIL2/4) in synovial fibroblasts. The differential regulation of co-expression of piRNAs in RASF and the induction of piRNA/Piwi-proteins by innate immune stimulators suggest a role in inflammatory processes.

## Introduction

Rheumatoid arthritis (RA) is a chronic autoimmune disease causing joint destruction as well as systemic inflammation.[1] Synovial fibroblasts (SF) are key effector cells in the pathogenesis of RA.[2] They produce pro-inflammatory cytokines, chemokines and matrix degrading enzymes attracting inflammatory cells to the joints and destroying the cartilage. RASF show an activated phenotype possibly due to epigenetic modifications such as DNA hypomethylation and derepression of transposons (e.g. LINE-1).[3, 4]

PIWI-interacting RNAs (piRNA; 24-32nt) build complexes with PIWIL (P-element induced wimpy testis like) proteins, members of the Argonaute family. There are four PIWIL proteins in humans (PIWIL1-4), which bind approximately 23`439 known piRNAs. [5] The PIWI/piRNA ribonucleoprotein complex regulates gene expression through specific recognition of the target gene by the base complementarity with the piRNA followed by target RNA degradation or the recruitment of chromatin modifying enzymes by the PIWIL protein. PIWI/piRNA complexes have a specific function in maintaining genome stability through silencing of transposons.[6] PIWIL proteins and piRNAs are known to be highly expressed in germline and cancer cells and the correlations of PIWIL and piRNA expression levels with patient prognosis was described in different cancer types.[7–10] Experimental manipulation of PIWI/piRNA expression both *in vitro* and *in vivo* influences growth, invasion and migration of the cancer cells.[7, 11–13] In addition, recent studies have shown a possible role of piRNAs in somatic cells, including the regulation of protein coding genes.[14–16] piRNAs can act in this context similarly to microRNAs by binding to the 3'UTR of messenger RNA and inducing its degradation.[17] MicroRNAs (e.g. miR-124a, -146a, -155 and -203) are known to be deregulated in RASF and to contribute to their above mentioned activated phenotype.[18] To the best of our knowledge, piRNA expression in RASF has not been studied so far.

The aim of our study was to 1) evaluate the expression of PIWIL1-4 proteins; 2) assess the expression of piRNAs and 3) study the regulation and function of the PIWI/piRNA system in RASF.

## Methods

### Synovial tissues and synovial fibroblast cell cultures

Synovial tissues were obtained from patients with RA diagnosed according to the 1987 American College of Rheumatology criteria for classification of RA,[19] who underwent joint reconstruction surgery (n = 32). Synovial tissues from patients with osteoarthritis (OA, degenerative disease) served as controls (n = 27). The study was approved by the Kantonale Ethikkommission der Kanton Zurich, with the approval numbers 475 and 515. All patients provided a written informed consent and the study was approved by the local Ethics committee. SF cultures were established by dispase digestion of synovial tissue as described elsewhere.[20] Cells were cultured in Dulbecco’s modified Eagle’s medium supplemented with 10% fetal calf serum. SF of passages 4–8 were used for all experiments. RASF and OASF were stimulated or not for 24 hours with Poly(I:C) (10ug/ml; Invivogen), LPS (100ng/ml; List Biological Laboratories) or TNFα (10 or 100ng/ml; R&D) alone or in combination with IL1β (1ng/ml; R&D).

### RNA isolation, reverse transcription, and Real-time polymerase chain reaction (PCR)

Cells were lysed in QIAzol lysis reagent, and total RNA was isolated using a miRNeasy Mini kit, including a DNA digestion step using RNase-free DNase (Qiagen). Total RNA was reverse transcribed using random hexamers and MultiScribe reverse transcriptase (Applied Biosystems). Non–reverse-transcribed (NRT) samples served as negative controls. Real-time PCR was performed using SYBR Green Master Mix or TaqMan® Gene Expression Master Mix (Applied Biosystems). PCR was performed using the 7500 Real-Time PCR System (Applied Biosystems). Relative expression of messenger RNA (mRNA) was calculated by the comparative threshold cycle method, where dCt = Ct (RNA of interest) − Ct (housekeeping gene) and therefore higher dCt values represent lower expression. Primer sequences were as follows: for PIWIL2, forward 5′-AAT-GCT-TCC-ATC-AGG-TAG-AGG-C-3′ and reverse 5′-TGT-CCT-TGC-GTA-CCA-GAT-TAG-C-3′; for PIWIL3, forward 5′-AGA-CAC-ATT-TAA-CAA-ATC-AGA-TGG-C-3′ and reverse 5′-TTC-TTT-ATG-TTG-CTG-CCT-GTA-GTA-G-3′; for PIWIL4, forward 5′-ACA-CGT-CTC-AGT-CCT-TCA-GG-3′, reverse 5′-AGC-GAG-CAT-TGG-TAT-TCC-TCT-G-3′; and for LINE1 ORF1, forward 5′-CAA-AGG-AAA-GCC-CAT-CAG-AC-3′, reverse 5′-GTA-GGG-TTT-CTG-CCG-AGA-GAT-3′. PIWIL1 gene was detected by TaqMAN assay (ID Hs01041737_m1, Applied Biosystems). HPRT1 (forward 5′-ATG-GAC-AGG-ACT-GAA-CGT-CTT-G-3′, reverse 5′-GGC-TAC-AAT-GTG-ATG-GCC-TC-3′) was used as an endogenous control.

### piRNA analysis by small RNA sequencing

Total RNA from RASF and OASF (n = 9 each, see Table 1 for patients characteristics) was isolated with the miRNeasy Mini kit and treated with RNase-free DNase (Qiagen). The RNA quality was assured using the Agilent RNA 6000 Nano kit with Agilent 2100 Bioanalyser instrument (Agilent Technologies). RNA with RNA integrity number (RIN) values ≥9.5 was considered acceptable for the study. Small RNA library preparations and sequencing using HiSeq2500 (Illumina Inc., CA) were performed at the Functional Genomic Center Zurich according to the Illumina TruSeq® Small RNA protocol. After removal of the adaptor sequences the small RNA-seq reads were mapped to the human genome by the alignment program Bowtie [21] and aligned to the piRNABank–database of 23`439 known human piRNA sequences.[5] Reads Per Kilobase per Million mapped reads (RPKM) of piRNAs identified in RA were compared to RPKM in OA using the SARTools pipeline based on edgeR package.[22] piRNAs were considered differentially expressed when the adjusted p-value was ≤ 0.05.

**Table 1:** Clinical data of patients–Small RNA sequencing in SF

Nr.	Dg.	Joint	Sex	Age	CRP	VAS	RA duration	RF	ACPA	DAS 28
SHK 303	OA	knee	f	72	6.4	0				
SHK 335	OA	knee	m	65	0.2	NA				
SHK 356	OA	knee	f	61	21.2	5				
SHK 350	OA	shoulder	f	89	2.5	NA				
SHK 384	OA	shoulder	f	68	1.2	9				
SHK 385	OA	shoulder	f	78	2.2	9				
SHK 345	OA	thumb	f	55	<8	NA				
SHK 400	OA	thumb	f	61	<5	NA				
SHK 343	OA	thumb	m	68	NA	NA				
SHK 196	RA	knee	f	54	46.7	1	16	1	1	NA
SHK 280	RA	knee	f	71	17.4	5.6	51	1	1	2
SHK 368	RA	knee	f	64	1.7	5	7	1	1	1.9
SHK 341	RA	shoulder	f	73	1.6	4	27	1	NA	1.3
SHK 352	RA	shoulder	f	69	8	5	39	1	NA	1.8
SHK 377	RA	shoulder	f	79	16.3	10	33	1	NA	3.9
SHK 316	RA	finger	f	52	NA	1	21	1	1	NA
SHK 318	RA	finger	f	69	NA	0	50	NA	NA	NA
SHK 379	RA	finger	f	78	26	0	23	1	1	NA

Age and RA duration in years; CRP in mg/l,VAS = Visual analogue scale for pain, 0-10cm; RF/ACPA 1 = positive

### Analysis of piRNAs by RT-PCR

The cDNAs were generated with TaqMan®MicroRNA Reverse Transcription Kit and a Custom designed piRNA-specific primer, followed by Real-time PCR with Custom designed piRNA TaqMan® probes and TaqMan Universal PCR Master Mix as recommended by the manufacturer (Applied Biosystems). We have measured the expression of piR-823, piR_4135, piR_16659 and piR-16735 [piRNA Bank[5]: hsa_piR_000823, hsa_piR-004135, hsa_piR_016659 and hsa_piR_016735]. RNU6B and RNU44 were employed as housekeeping genes.

### Western blotting

RASF were trypsinized and nuclear and cytoplasmic extracts prepared with NC-PER Nuclear and Cytoplasmic Extraction Reagents (Thermo Scientific) according to the manufacturers protocol. Proteins were separated by sodium dodecyl sulfate polyacrylamide gel electrophoresis and transferred to polyvinylidene fluoride (PVDF) membrane (Amersham Hybond^TM^ P; GE Healthcare). Membranes were blocked with 5% milk in TBS-T and incubated overnight at 4°C with rabbit anti-human PIWIL4 (PA5-31448; Thermo Scientific), rabbit anti-human PIWIL2 (sc-67303; Santa Cruz), rabbit anti-human Lamin B1 (ab16048; Abcam) and mouse anti-human α-tubulin (ab7291; Abcam) antibodies in 5% milk. HRP-labeled species-specific secondary antibodies (Jackson Immuno Research) were used and signals were detected using enhanced chemiluminescence Western Bright^TM^ ECL (Advansta) and the Fusion FX imager (Vilber Lourmat). The Alpha Imager software system (Alpha Innotech) was used to analyse the results.

### Immunofluorescence

Paraffin-embedded synovial sections were incubated with monoclonal mouse antihuman PIWIL4 (MA5-17151, Thermo Scientific) antibodies or normal mouse IgG1 (Dako) followed by addition of Alexa Fluor 555-conjugated goat antimouse IgG (Invitrogen). Slides were placed in ProLong Gold antifade reagent mounting medium with 4',6-diamidino-2-phenylindole (Invitrogen). Images were collected using a confocal microscope (Imager.21; Zeiss). The background fluorescence level was set with the negative controls, and images were analysed using AxioVision Rel. 4.8. image analysis software (Zeiss).

### Silencing of PIWIL2 and 4

RASFs were transfected with small interfering RNAs (siRNA, 100nM) using Lipofectamine 2000 (Invitrogen) according to the manufacturer´s instructions. Twenty-four hours after transfection, the medium was replaced and 48 hours after transfection, cells were harvested for total RNA isolation and functional tests. Cell lysates for Western blot were prepared 72 hours after transfections. Following siRNAs (Qiagen) were used: siPIWIL2 (Hs_PIWIL2_5), siPIWIL4 (Hs_PIWIL4_2, Hs_PIWIL4_6 and Hs_PIWIL4_8) and negative control (AllStars Negative Control siRNA).

### Analysis of cell proliferation

Proliferation of SF after silencing PIWIL2 and 4 was analysed with the BrdU (Bromodeoxyuridine) proliferation assay (Roche) according to the manufacturers instructions.

## LINE-1 methylation assay

To determine the LINE-1 methylation we used the Global Methylation LINE-1 Kit (Active Motif) according to manufacturer’s instructions. RASF treated for 1 week with 5-azacytosine (1μM; Sigma) served as a positive control.

### Statistical analysis

The statistical analysis was performed using the GraphPad Prism 5.0 software. Data were analysed by parametric (paired 2-tailed t-test) or nonparametric (Wilcoxon's matched pairs signed rank test) statistical tests as appropriate. Values are presented as mean ± SEM. P values less than 0.05 were considered significant.

## Results

### PIWIL2 and PIWIL4 genes are transcribed in synovial fibroblasts

**Fig 1:**
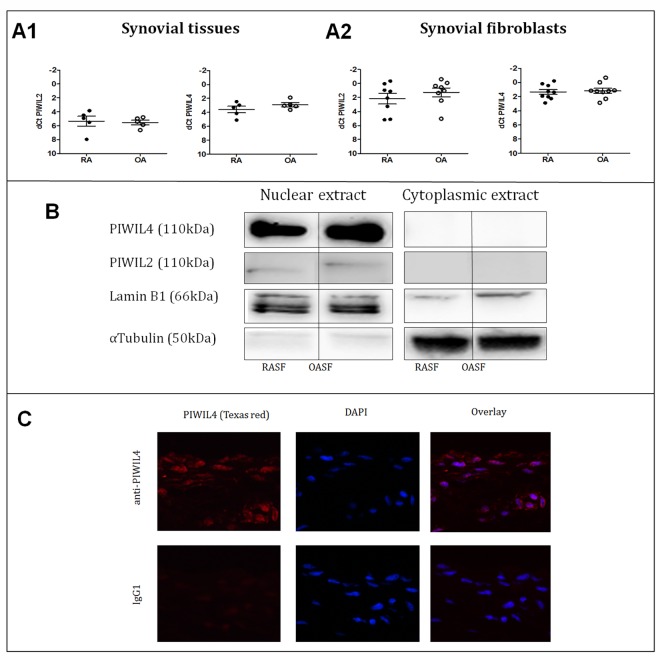
Expression of PIWIL genes A1. mRNA expression of PIWIL2 and PIWIL4 in synovial tissues (ST) from RA and OA patients. Mean dCt PIWIL2 in RAST 5.3 and in OAST 5.5; mean dCt PIWIL4 in RAST 3.6 and in OAST 2.9. PIWIL1 and PIWIL3 were not expressed. **A2.** mRNA expression of PIWIL2 and PIWIL4 in isolated synovial fibroblasts (SF) from RA and OA patients. Mean dCt PIWIL2 in RASF 2.2 and in OASF 1.3; mean dCt PIWIL4 in RASF 1.3 and in OASF 1.2. PIWIL1 and PIWIL3 were not expressed. **B.** PIWIL4 protein was detected by Western blot in both RASF and OASF in the nucleus, but not in the cytoplasm. PIWIL2 expression was weak. **C.** Immunofluorescence has shown the presence of PIWIL4 protein predominantly in the cell nucleus and in the perinuclear regions (representative example of a RASF culture).

To investigate whether the PIWI/piRNA system is present in the synovium, we first measured the expression of the PIWIL1-4 mRNA in RA and OA synovial tissues. Transcripts for PIWIL2 and PIWIL4 were expressed similarly in RA and OA synovial tissues (mean PIWIL2 dCt±SEM in RA 5.3±0.7 and in OA 5.5±0.3; mean PIWIL4 dCt±SEM in RA 3.6±0.5 and in OA 2.9±0.3, Fig 1A1), while mRNA for PIWIL1 and PIWIL3 were not detectable. Synovial tissue is composed by multiple cell types (SF, inflammatory infiltrates, endothelial cells and others), and SF themselves are recognized as key players in pathogenesis of RA. We measured the expression of PIWIL1-4 mRNA in cultured SF and could detect similar high expression of PIWIL2 and PIWIL4 mRNA in cells from both RA and OA patients (mean PIWIL2 dCt±SEM in RA 2.2±0.8 and in OA 1.3±0.6; mean PIWIL4 dCt±SEM in RA 1.3±0.3 and in OA 1.2±0.4).(Fig 1A2)

### PIWIL4 protein is expressed in cell nuclei

At the protein level, we detected strong expression of PIWIL4 in nuclear extracts from RASF and OASF on Western blots, while the bands of PIWIL2 were weaker in both RASF and OASF. Neither PIWIL2 nor PIWIL4 protein were found in the cytoplasmic extract. (Fig 1B) For the following analysis, we focused on PIWIL4 and performed the immunofluorescence analysis in RASF, which showed PIWIL4 protein expression in the cell nuclei as well as in the perinuclear region (Fig 1C).

### Piwi-interacting RNAs are expressed in synovial cells

**Fig 2:**
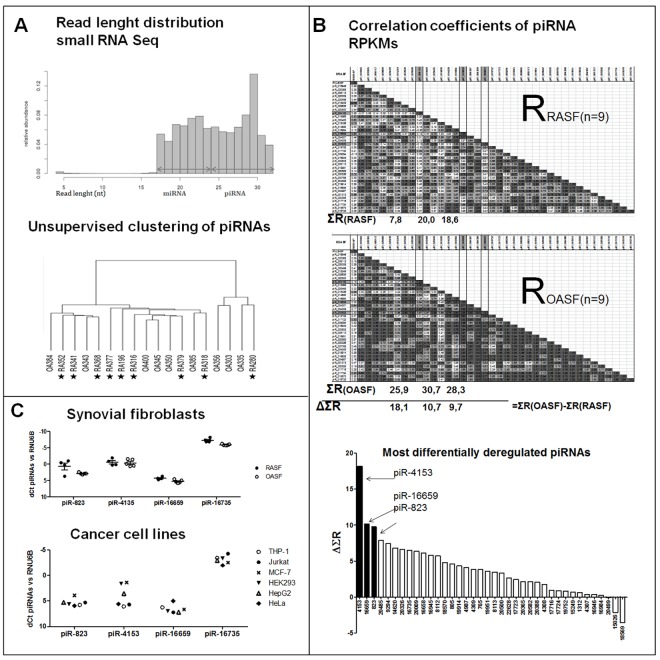
Expression of PIWI-interacting RNAs **A.** Small RNA Illumina sequencing results: read length distribution in all synovial fibroblasts (SF) peaked at 23 and 30nt. piRNAs did not cluster 9 RASF and 9 OASF. **B.** Correlation matrix of piRNAs and RNU6-6P (endogenous control) in 9 RASF and 9 OASF. RPKM of single piRNAs were correlated with each other, the correlation coefficient R for each pair is shown. Only piRNAs with mean RPKM ≥500 (n = 38) were included in this analysis. Strong positive correlations (0.75≤R) are highlighted with dark grey and 0.5≤R<0.75 are light grey. In RASF was this regulation of piRNA co-expression less tight compared to OASF. SUMA (Σ) R was counted for each piRNA in 9 RASF and in 9 OASF. Regulation of piRNAs in RASF versus OASF was compared by counting delta (Δ) of the ΣR(OASF) and ΣR(RASF). The least tightly regulated piRNAs were piR-4153, piR-16659 and piR-823 (ΔΣR 18.1, 10.7 and 9.7). **C.** In additional 5 RASF and 5 OASF, piRNAs were expressed as follows (mean dCt versus RNU6B RA/OA): piR-823 (0.6/2.8), piR-4153 (-0.6/-0.4), piR-16659 (4.3/5.2) and piR-16735 (-7.3/-5.9). Similar expression were also detected in cancer cell lines (THP-1, Jurkat, MCF-7, HEK293, HepG2 and HeLa).

Next, we sequenced 9 RASF and 9 OASF small RNA libraries using the Illumina platform. Reads length distribution peaks at 22nt and 29nt, confirming the presence of both miRNAs and piRNAs. Up to 300 piRNAs were found to be expressed in all 9 RASF and OASF. The most expressed piRNA, piR-16735, covered 20% of all piRNA reads, while the next 3 piRNAs (piR-18570, -17724 and -20388) each covered about 5% of all piRNA reads. The analysis of RNA seq count data did not show any significantly differentially expressed piRNAs in RASF compared to OASF. Unsupervised clustering of piRNA could not distinguish between the groups of RASF and OASF.(Fig 2A and S3 Appendix and S4 Appendix)

### Coexpression of piRNAs in RA and OA synovial fibroblasts

The RNA sequencing data (RPKM) suggested some mechanism of co-expression or co-regulation of piRNAs in SF. To investigate this issue, we correlated RPKMs of single piRNAs in the 9 RASF and 9 OASF between each other and created correlation matrixes showing the Pearson´s correlation coefficients R for each pair of piRNAs. All coefficients were mostly positive, as shown in the Fig 2B and S1 Appendix. Furthermore, we have calculated the ΔR = R(OASF)-R(RASF) for the single piRNAs and then summed all ΔR for each piRNA. According to this analysis, piRNAs were more tightly regulated in OASF than in RASF (ΔR are mostly positive) and the 3 least tightly regulated piRNAs in RASF were piR-823, -4153 and -16659 (Fig 2B and Supplementary Figs 1 and 2). Expression levels of those piRNAs were comparable in SF and in different cancer cell lines; piR-16735 was the most expressed piRNA, followed by piR-4153, piR-823 and the least expressed piR-16659 (Fig 2C and S2 Appendix).

### TLR-ligands and proinflammatory cytokines increase expression of PIWIL4

To understand, whether PIWIL4 is regulated by the inflammatory process in the RA and OA joints, we measured PIWIL4 expression in RASF and OASF stimulated with TLR-ligands LPS and Poly(I:C) or with the proinflammatory cytokines IL1β in combination with TNFα. Levels of PIWIL4 mRNA were enhanced by Poly(I:C) RASF/OASF 3.2-fold (p = 0.0017)/3.4-fold (p = 0.0133); LPS 2.1-fold (p = 0.0262)/2.6-fold (p = 0.0249) and TNFα in combination with IL1β 1.9-fold (p = 0.0035)/ 1.7-fold (p = 0.0075).

Levels of PIWIL2 mRNA were enhanced only by TNFα in combination with IL1β in RASF/OASF 1.9-fold (p = 0.0169)/1.7-fold (p = 0.0079). Otherwise PIWIL2 mRNA levels were not significantly changed after stimulation with LPS and Poly IC.

**Fig 3:**
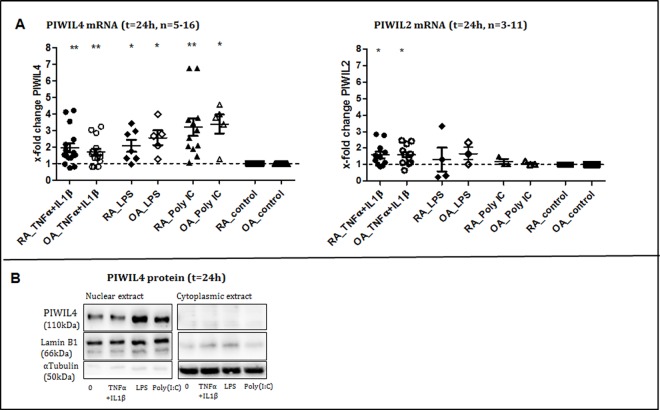
Regulation and function of PIWIL/piRNAs **A.** Levels of PIWIL4 mRNA in RASF and OASF were enhanced by stimulations with proinflammatory cytokines and TLR ligands. TNFα in combination with IL1β induced PIWIL4 mRNA in RASF/OASF 1.9-fold (p = 0.0035)/ 1.7-fold (p = 0.0075); LPS 2.1-fold (p = 0.0262)/2.6-fold (p = 0.0249) and Poly(I:C) 3.2-fold (p = 0.0017)/3.4-fold (p = 0.0133). ● = RASF, ○ = OASF **B.** On the protein level we confirmed upregulation of PIWIL4 after stimulation with TLR-ligands in nuclear extract of RASF, there was no PIWIL4 detectable in the cytoplasmic extract.

At the protein level by Western blot we detected a similar induction of PIWIL4 expression in the nuclear extract, while in the cytoplasmic extract PIWIL4 protein was not detectable.(Fig 3B).

**Fig 4:**
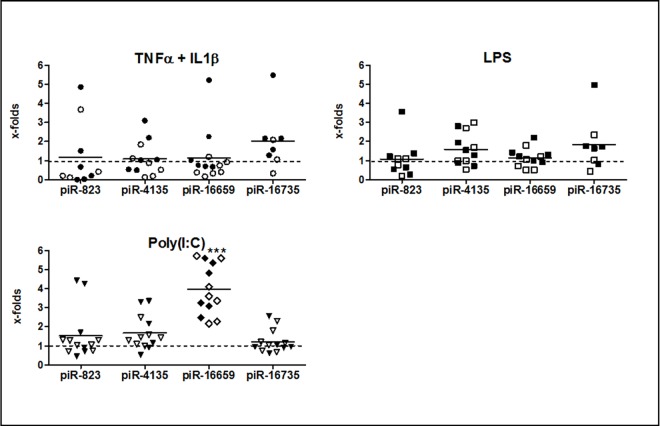
Regulation of piRNAs by inflammatory stimulators in RASF and OASF Expression of piR-16659 was 4-fold increased upon Poly(I:C) stimulation (p = 0.001) in RASF an OASF, levels of other piRNAs were not significantly changed 24 hours after stimulation with TNFα + IL1β, LPS or Poly(I:C). ● = RASF, ○ = OASF

Expression of piR-16659 was 4/3.83-fold increased upon Poly(I:C) stimulation in RASF and OASF (p = 0.0069/0.0019), otherwise the piRNA levels were not significantly changed 24 hours after stimulation with TNFα and IL1β, LPS or Poly(I:C) (Fig 4).

### PIWIL/piRNA system is probably not involved in the regulation of RASF proliferation

**Fig 5:**
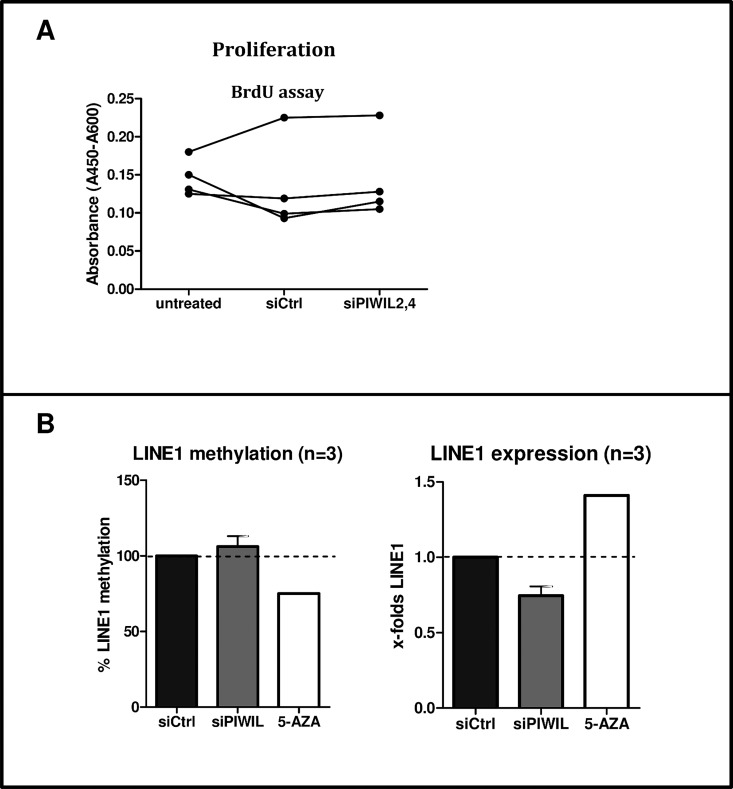
Effects of PIWIL silencing on proliferation and methylation **A.** Cell proliferation in RASF (n = 4) silenced for PIWIL2 and 4 genes. **B.** PIWIL2 and PIWIL4 silencing did not change expression or methylation of the LINE-1 retrotransposons (n = 3; mean LINE1 methylation 106%, mean mRNA expression 0.74-fold). RASF treated for 1 week with the DNA demethylation drug 5-azacytidine (5-AZA) were used as positive control.

In cancer, silencing of PIWIL4 decreases proliferation. We evaluated the cell proliferation in RASF (n = 4) silenced for PIWIL2 and 4 genes after 24 hours. We have not observed any change of cell proliferation as detected by the BrdU assay.(Fig 5A)

### PIWIL4 does not regulate methylation or expression of LINE-1

piRNAs help to maintain the integrity of the genome by silencing the transposons.[6] To investigate whether this mechanism might play a role in RA, we have determined LINE-1 methylation and expression in RASF after silencing of PIWIL2 and 4. Transient silencing of PIWIL2 and 4 did neither change the LINE-1 methylation nor its RNA expression, which was opposite to the treatment with 5-AZA.(Fig 5B).

## Discussion

In this study we demonstrate: 1) Expression of PIWIL2 and 4 protein in RA and OASF. 2) Expression of up to 300 piRNAs in RA and OASF at similar levels. The regulation but not the expression levels of piRNA-823, -4153 and -16659 were different in RASF compared to OASF. 3) Regulation of PIWIL2 and 4 but not piRNAs proinflammatory cytokines in RASF and OASF. 4) Induction of piR-16659 by the TLR-ligand Poly IC in RASF and OASF and 5) No effect of silencing of PIWIL genes on the proliferation of RASF and the methylation/expression of LINE-1.

Our finding that PIWIL4 protein is expressed in SF is in agreement with a previous study by Sugimoto et al., who reported that the PIWIL4 gene is expressed in various somatic tissues.[23] The number of piRNAs which we detected in RASF is comparable to 273 piRNAs detected in somatic tissues by Matrinez et al.[24]

piRNA expression levels mostly correlate positively with each other suggesting co-regulation of the piRNA expression by some common mechanism. This co-regulation is less tight in RASF than in OASF, particularly for piR-4153, piR-16659 and piR-823. This suggests the presence of a regulatory factor in OASF, which might be missing in RASF. Common regulation of piRNAs could be partially explained by their transcription in clusters.[14] However piR-4153, -16659 and -823 are coded on different chromosomes. Further studies are needed to clarify, whether the piRNA deregulation is more involved in the activation of RASF or if it is only a bystander effect.

We also describe an additional regulatory mechanism of the PIWI/piRNA system by proinflammatory cytokines and TLR-ligands in addition to the hormonal regulation reported by others.[25, 26]

In cervical cancer cells, upregulation of PIWIL4 promotes cell proliferation and decreases apoptosis.[11] We did not observe any changes in the proliferation of RASF after silencing PIWIL4; a possible reason is that the proliferation rate of SF is low in comparison to that of cancer cells.

The upregulation of PIWIL4 and partially PIWL2 by inflammatory cytokines and innate immune stimulators in RASF and OASF suggests that these genes might be involved in signaling pathways for host defense and inflammation. Further, the significant induction of piR-16659 by Poly(I:C) indicates a general role in Toll-like-receptor (TLR)3 mediated signaling- e.g. during viral infaction.

In our study, the transient silencing of PIWIL2 and 4 did not change the methylation and expression of LINE-1 in RASF. This is interesting in light of the fact that LINE-1 is known to be deregulated in RASF [3] and the PIWIL/piRNA system is believed to have a main function in silencing of transponsable elements.[6] However there is another system to control LINE-1 expression, namely TREX-1 which has been found to be deficient in RASF,[27] suggesting a redundancy in LINE-1 silencing mechanisms with apparently less importance of the PIWIL/piRNA system in RASF.

One limitation of the study is the use of patient material from surgery of patients with longstanding disease (mean RA duration 25 years (29 years in RNA seq, 23 years in functional experiments). The use of patient material from arthroscopy of patients with early disease could give more pathogenesis relevant information. Another limitation of this study is the lack of identification of a functional consequence of the PIWI and piRNA expression we described.

## Conclusion

To summarise our findings, we detected a new class of regulatory RNAs and their binding partners in RA and OA synovial fibroblasts. Up to 300 PIWI-interacting RNAs, which build complexes with PIWIL proteins, are transcribed in SF. The deregulation of some piRNAs, as well as the fact that they can be upregulated by Poly(I:C) suggest that the PIWI/piRNA pathway is involved in the innate immunity and may contribute to the pathogenesis of RA.

## Supporting Information

S1 Appendix Differentially correlated piRNAs in RASF vs OASF Analysis of differentially regulated piRNAs in 9 RASF versus 9 OASF. In the correlation matrix are shown ΔR = R(OASF)-R(RASF) for each piRNA. With orange are highlighted piRNA correlations, which are weaker in RASF compared to OASF (ΔR≤-0,5). In the last line is the ΣΔR for each piRNA given, the three less tightly regulated piRNAs in RASF versus OASF are piR-4153, piR-16659 and piR-823.Correlation coefficient (R) = 1 means 100% positive correlation (the more of A, the more of B) = 0 means no correlation = -1 means 100% negative correlation (the more of A, the less of B)
(TIF)

S2 Appendix Interrelationship between pi-RNAs (n = 38) in RASF and OASF. A. Mean of the correlation coefficients calculated for each piRNA in relation with the 37 others. RASF showed a highly significant (p < 0.001) lower mean than OASF. This suggests that the expression of piRNAs are differently regulated in RASF than in OASF. B. The correlation coefficients obtained by given piRNAs were compared between RASF and OASF; logarithmic p-values (two tailed t-test) were calculated for each pi-RNA. This suggested that piwi-4153, -16659 and -823 are less tightly regulatedin RASF than in OASF. C. List of piRNAs with logarithmic p-values lower than -3 (i.e., with p < 0.001).
(TIF)

S3 Appendix RNA sequencing data (RPKM) Small RNA library preparations and sequencing using HiSeq2500 (Illumina Inc., CA) were performed at the Functional Genomic Center Zurich. This table shows human piRNA sequences Reads Per Kilobase per Million mapped reads (RPKM) identified in RASF and OASF.
(XLSX)

S4 Appendix Statistical report for the sequencing data analysis Reads Per Kilobase per Million mapped reads (RPKM) of piRNAs in RA were compared to RPKM in OA using the SARTools pipeline based on edgeR package.
(PDF)

S5 Appendix All the clinical data from the patient samples used in this study We listed all available clinical patient data and indicated the Experiments/Figure numberes, where the samples were used.
(XLSX)
