# Supplementary figures and images for: Expression and Regulation of PIWIL-Proteins and PIWI-Interacting RNAs in Rheumatoid Arthritis
Source: PLoS One. 2016 Nov 28;11(11):e0166920. doi: 10.1371/journal.pone.0166920 (PMC5125648; doi:10.1371/journal.pone.0166920)

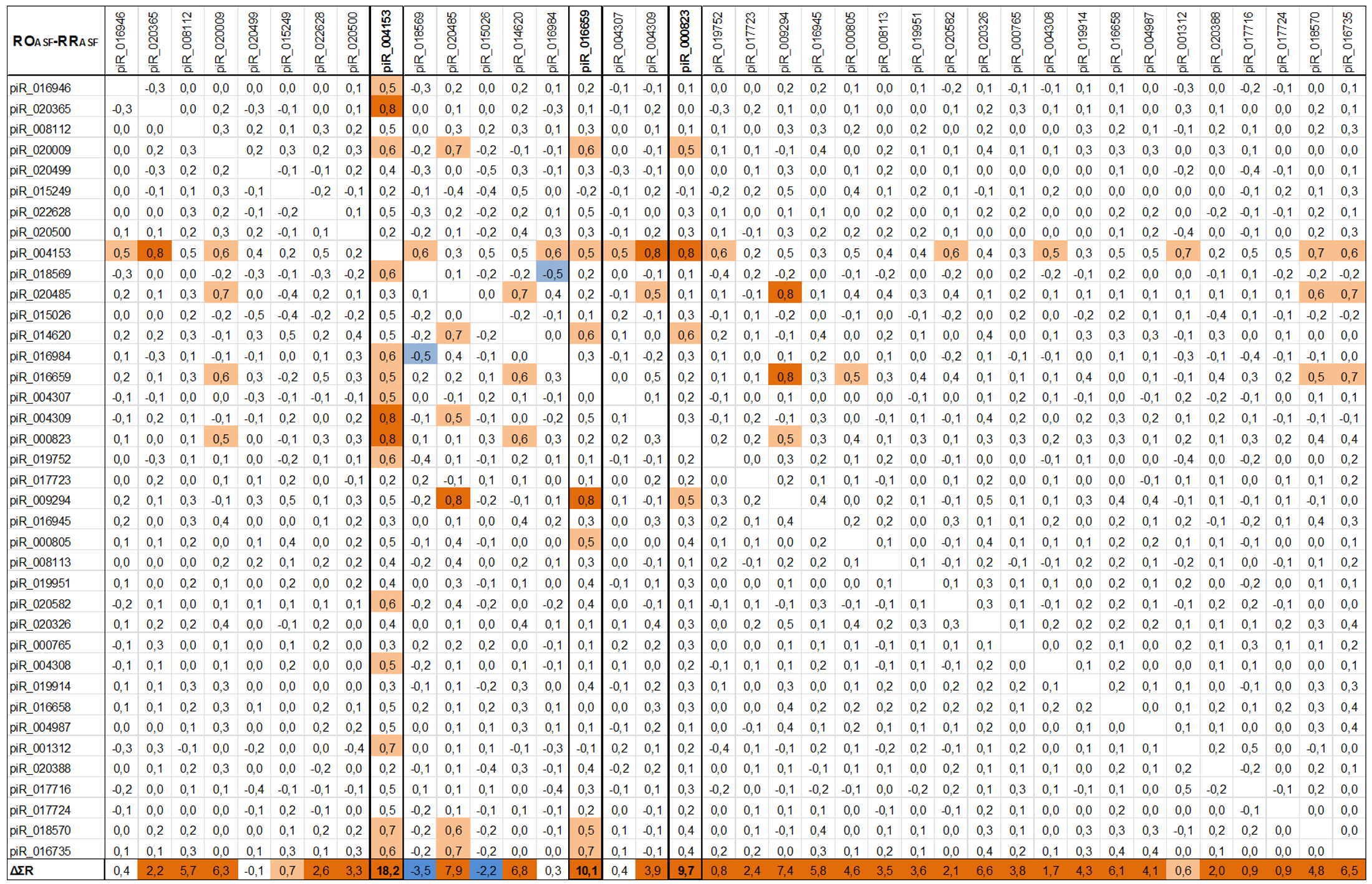

Supplement: S1 Appendix — Analysis of differentially regulated piRNAs in 9 RASF versus 9 OASF. In the correlation matrix are shown ΔR = R(OASF)-R(RASF) for each piRNA. With orange are highlighted piRNA correlations, which are weaker in RASF compared to OASF (ΔR≤-0,5). In the last line is the ΣΔR for each piRNA given, the three less tightly regulated piRNAs in RASF versus OASF are piR-4153, piR-16659 and piR-823.Correlation coefficient (R) = 1 means 100% positive correlation (the more of A, the more of B) = 0 means no correlation = -1 means 100% negative correlation (the more of A, the less of B) (TIF) [file pone.0166920.s001.tif]

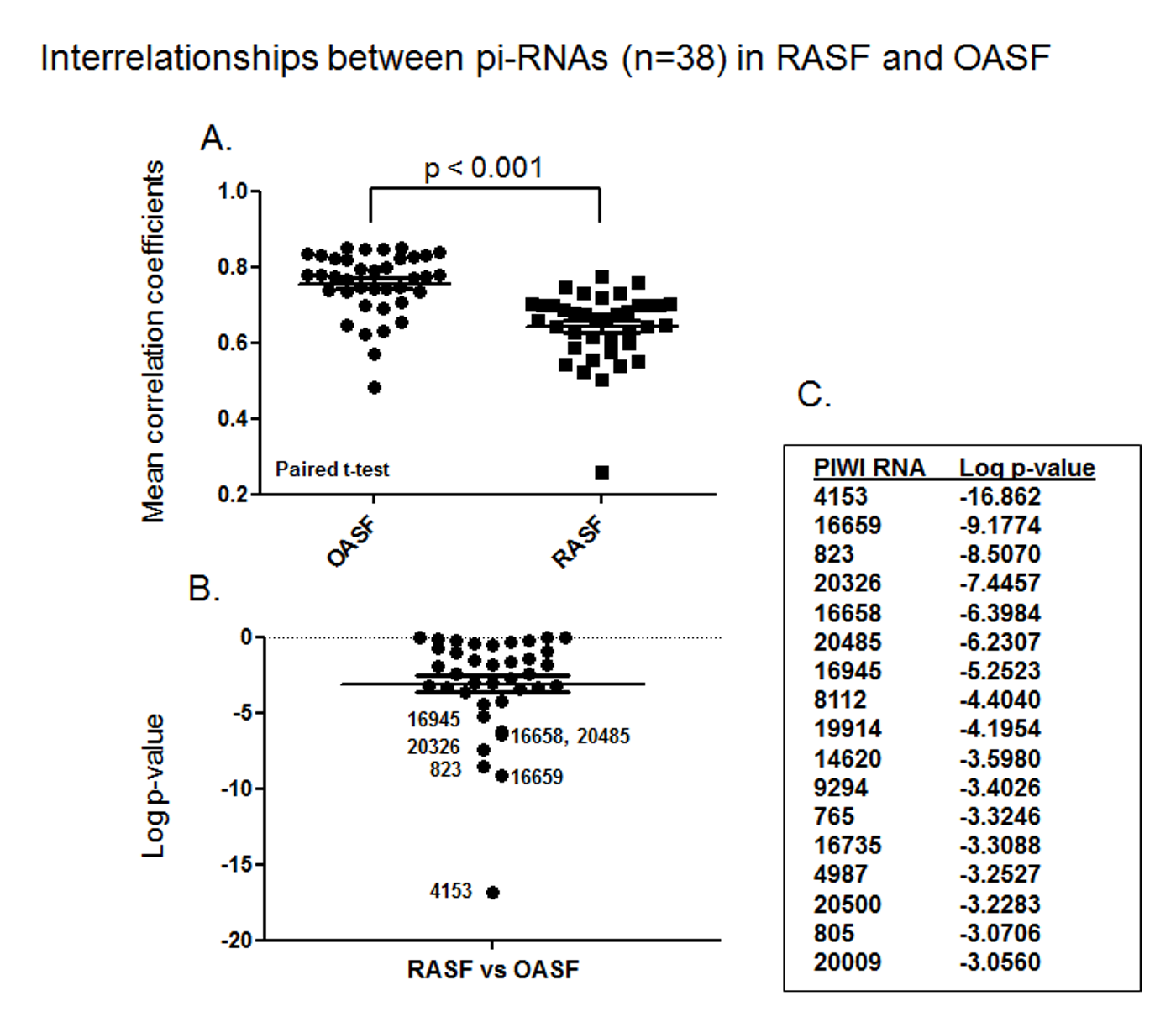

Supplement: S2 Appendix — A. Mean of the correlation coefficients calculated for each piRNA in relation with the 37 others. RASF showed a highly significant (p < 0.001) lower mean than OASF. This suggests that the expression of piRNAs are differently regulated in RASF than in OASF. B. The correlation coefficients obtained by given piRNAs were compared between RASF and OASF; logarithmic p-values (two tailed t-test) were calculated for each pi-RNA. This suggested that piwi-4153, -16659 and -823 are less tightly regulatedin RASF than in OASF. C. List of piRNAs with logarithmic p-values lower than -3 (i.e., with p < 0.001). (TIF) [file pone.0166920.s002.tif]
