# Supplementary material for: Expression and Regulation of PIWIL-Proteins and PIWI-Interacting RNAs in Rheumatoid Arthritis
Source: PLoS One. 2016 Nov 28;11(11):e0166920. doi: 10.1371/journal.pone.0166920 (PMC5125648; doi:10.1371/journal.pone.0166920)
Supplement: S4 Appendix — Reads Per Kilobase per Million mapped reads (RPKM) of piRNAs in RA were compared to RPKM in OA using the SARTools pipeline based on edgeR package. (PDF) [file pone.0166920.s004.pdf]

Statistical report of project stats-edgeR: pairwise comparison(s) of conditions with edgeR

Author: Your name

Date: 2015-10-05

The SARTools R package which generated this report has been developped at PF2 - Institut Pasteur by M.-A. Dillies and H. Varet (hugo.varet@pasteur.fr). Thanks to cite H. Varet, J.-Y. Coppee and M.-A. Dillies, *SARTools: a DESeq2- and edgeR-based R pipeline for comprehensive differential analysis of RNA-seq data*, 2015 (submitted) when using this tool for any analysis published.

Table of contents

1. Introduction
2. Description of raw data
3. Filtering low counts
4. Variability within the experiment: data exploration
5. Normalization
6. Differential analysis
7. R session information and parameters
8. Bibliography

1 Introduction

The analyses reported in this document are part of the stats-edgeR project. The aim is to find features that are differentially expressed between OA and RA. The statistical analysis process includes data normalization, graphical exploration of raw and normalized data, test for differential expression for each feature between the conditions, raw p-value adjustment and export of lists of features having a significant differential expression between the conditions.

The analysis is performed using the R software [R Core Team, 2014], Bioconductor [Gentleman, 2004] packages including edgeR [Robinson, 2010] and the SARTools package developed at PF2 - Institut Pasteur. Normalization and differential analysis are carried out according to the edgeR model and package. This report comes with additional tab-delimited text files that contain lists of differentially expressed features.

For more details about the edgeR methodology, please refer to its related publications [Robinson, 2007, 2008, 2010 and McCarthy, 2012].

2 Description of raw data

The count data files and associated biological conditions are listed in the following table.

label	files	group
OA384_04112013_SH_OA	OA384_04112013_SH_OA.txt	OA
356_RNA1_KN_OA	356_RNA1_KN_OA.txt	OA
OA385_04112013_SH_OA	OA385_04112013_SH_OA.txt	OA
400_RNA2_HA_OA	400_RNA2_HA_OA.txt	OA
335_RNA1_KN_OA	335_RNA1_KN_OA.txt	OA
303_RNA2_KN_OA	303_RNA2_KN_OA.txt	OA
345_RNA1_HA_OA	345_RNA1_HA_OA.txt	OA
350_RNA2_SH_OA	350_RNA2_SH_OA.txt	OA
343_06122013_HA_OA	343_06122013_HA_OA.txt	OA
318_RNA2_HA_RA	318_RNA2_HA_RA.txt	RA
379_RNA2_HA_RA	379_RNA2_HA_RA.txt	RA
352_RNA1_SH_RA	352_RNA1_SH_RA.txt	RA
341_RNA1_SH_RA	341_RNA1_SH_RA.txt	RA
377_RNA2_SH_RA	377_RNA2_SH_RA.txt	RA
368_RNA1_KN_RA	368_RNA1_KN_RA.txt	RA
316_06122013_HA_RA	316_06122013_HA_RA.txt	RA
RA196_04112013_KN_RA	RA196_04112013_KN_RA.txt	RA
280_RNA1_KN_RA	280_RNA1_KN_RA.txt	RA

Table 1: Data files and associated biological conditions.

After loading the data we first have a look at the raw data table itself. The data table contains one row per annotated feature and one column per sequenced sample. Row names of this table are feature IDs (unique identifiers). The table contains raw count values representing the number of reads that map onto the features. For this project, there are 515 features in the count data table.

	OA384_04112013_SH_OA	356_RNA1_KN_
hsa_piR_000043|gb|DQ569990|Homo	0	
hsa_piR_000045|gb|DQ569993|Homo	37	
hsa_piR_000291|gb|DQ570344|Homo	1	
hsa_piR_000354|gb|DQ570422|Homo	0	
hsa_piR_000402|gb|DQ570485|Homo	0	
hsa_piR_000441|gb|DQ570540|Homo	148	

Looking at the summary of the count table provides a basic description of these raw counts (min and max values, median, etc).

	OA384_04112013_SH_OA	356_RNA1_KN_OA	OA385_04112013_S
Min.	0	0	
1st Qu.	0	0	
Median	9	2	
Mean	267	107	
3rd Qu.	71	19	
Max.	28893	9756	

Figure 1 shows the total number of mapped reads for each sample. Reads that map on multiple locations on the transcriptome are counted more than once, as far as they are mapped on less than 50 different loci. We expect total read counts to be similar within conditions, they may be different across conditions. Total counts sometimes vary widely between replicates. This may happen for several reasons, including:

- different rRNA contamination levels between samples (even between biological replicates);
- slight differences between library concentrations, since they may be difficult to measure with high precision.

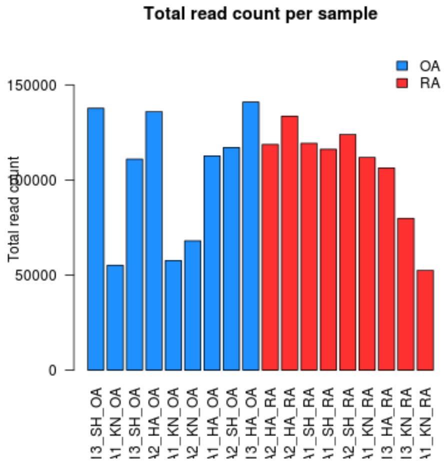

Figure 1: Number of mapped reads per sample. Colors refer to the biological condition of the sample.

Figure 2 shows the proportion of features with no read count in each sample. We expect this proportion to be similar within conditions. Features with null read counts in the 18 samples will not be taken into account for the analysis with edgeR. Here, 0 features (0%) are in this situation (dashed line).

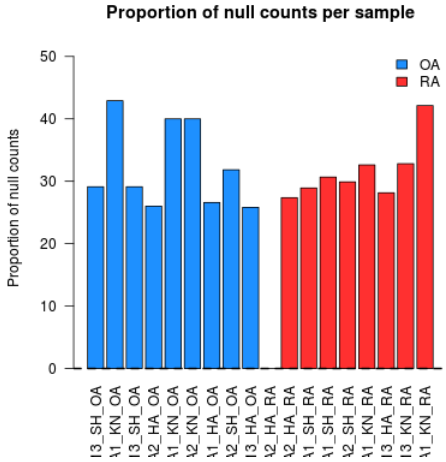

Figure 2: Proportion of features with null read counts in each sample.

Figure 3 shows the distribution of read counts for each sample. For sake of readability, $\log_2(\text{counts}+1)$ are used instead of raw counts. Again we expect replicates to have similar distributions. In addition, this figure shows if read counts are preferably low, medium or high. This depends on the organisms as well as the biological conditions under consideration.

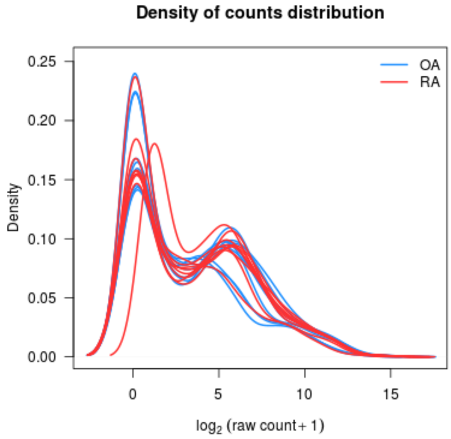

Figure 3: Density distribution of read counts.

It may happen that one or a few features capture a high proportion of reads (up to 20% or more). This phenomenon should not influence the normalization process. The edgeR normalization has proved to be robust to this situation [Dillies, 2012]. Anyway, we expect these high count features to be the same across replicates. They are not necessarily the same across conditions. Figure 4 and table 4 illustrate the possible presence of such high count features in the data set.

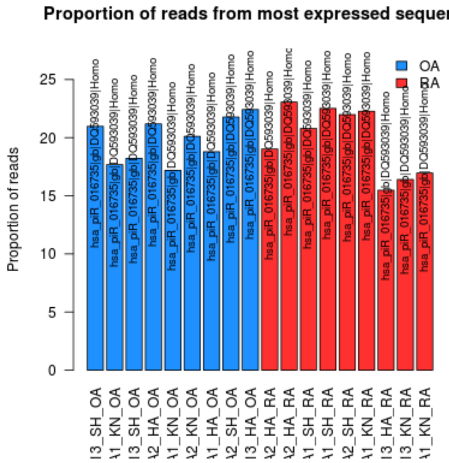

Figure 4: Percentage of reads associated with the sequence having the highest count (provided in each box on the graph) for each sample.

	OA384_04112013_SH_OA	356_RNA1_KN_
hsa_piR_016735|gb|DQ593039|Homo	20.98	[illegible]
hsa_piR_018570|gb|DQ595536|Homo	7.77	
hsa_piR_017724|gb|DQ594465|Homo	4.27	
hsa_piR_020388|gb|DQ598008|Homo	3.26	
hsa_piR_001312|gb|DQ571813|Homo	3.24	

We may wish to assess the similarity between samples across conditions. A pairwise scatter plot is produced (figure 5) to show how replicates and samples from different biological conditions are similar or different ($\log_2(\text{counts} + 1)$ are used instead of raw count values). Moreover, as the Pearson correlation has been shown not to be relevant to measure the similarity between replicates, the SERE statistic has been proposed as a similarity index between RNA-Seq samples [Schulze, 2012]. It measures whether the variability between samples is random Poisson variability or higher. Pairwise SERE values are printed in the lower triangle of the pairwise scatter plot. The value of the SERE statistic is:

- 0 when samples are identical (no variability at all: this may happen in the case of a sample duplication);
- 1 for technical replicates (technical variability follows a Poisson distribution);
- greater than 1 for biological replicates and samples from different biological conditions (biological variability is higher than technical one, data are over-dispersed with respect to Poisson). The higher the SERE value, the lower the similarity. It is expected to be lower between biological replicates than between samples of different biological conditions. Hence, the SERE statistic can be used to detect inversions between samples.

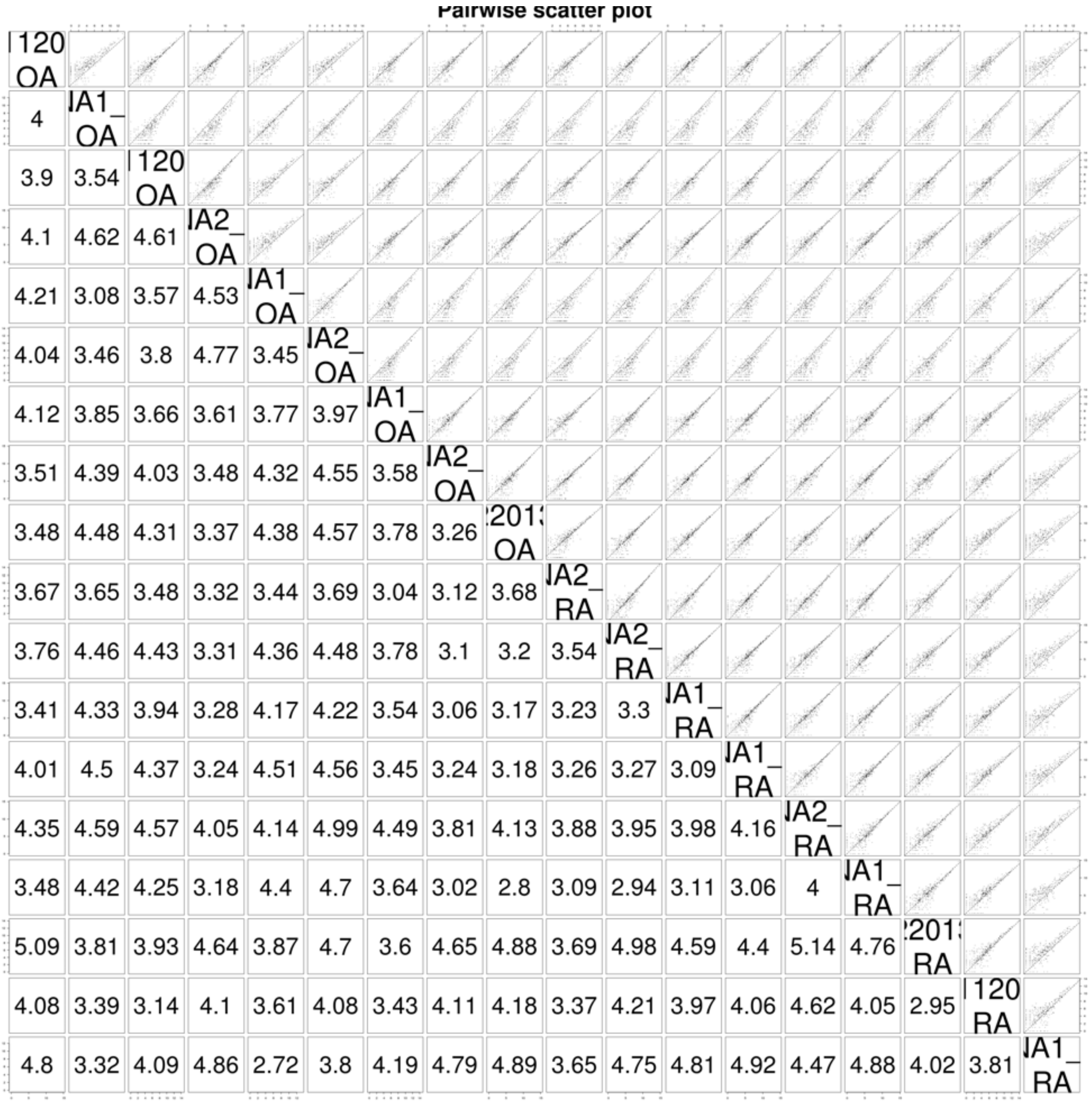

Figure 5: Pairwise comparison of samples.

3 Filtering low counts

edgeR suggests to filter features with null or low counts because they do not supply much information. For this project, 162 features (31.46%) have been removed from the analysis because they did not satisfy the following condition: having at least 1 counts-per-million in at least 9 samples.

4 Variability within the experiment: data exploration

The main variability within the experiment is expected to come from biological differences between the samples. This can be checked in two ways. The first one is to perform a hierarchical clustering of the whole sample set. This is performed after a transformation of the count data as moderated log-counts-per-million. Figure 6 shows the dendrogram obtained from CPM data. An euclidean distance is computed between samples, and the dendrogram is built upon the Ward criterion. We expect this dendrogram to group replicates and separate biological conditions.

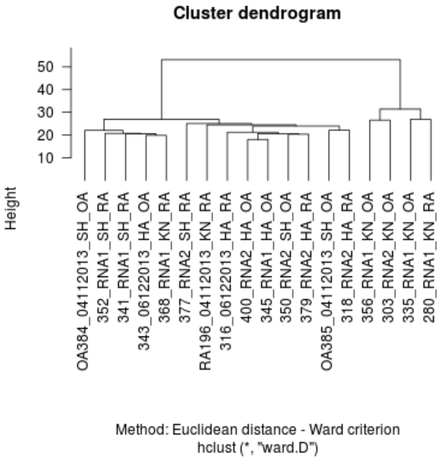

Figure 6: Sample clustering based on normalized data.

Another way of visualizing the experiment variability is to look at the first two dimensions of a multidimensional scaling plot, as shown on figure 7. On this figure, the first dimension is expected to separate samples from the different biological conditions, meaning that the biological variability is the main source of variance in the data.

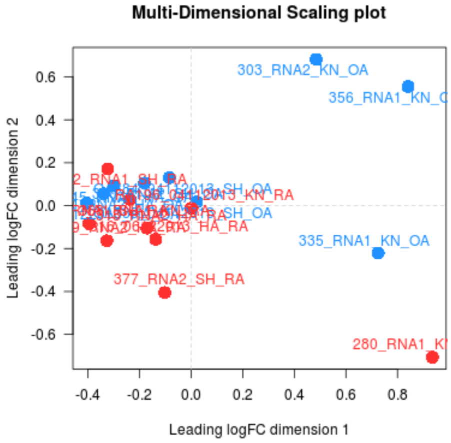

Figure 7: Multidimensional scaling plot of the samples.

5 Normalization

Normalization aims at correcting systematic technical biases in the data, in order to make read counts comparable across samples. The normalization proposed by edgeR is called Trimmed Mean of M-values (TMM) but it is also possible to use the RLE (DESeq) or upperquartile normalizations. It relies on the hypothesis that most features are not differentially expressed.

edgeR computes a factor for each sample. These normalization factors apply to the total number of counts and cannot be used to normalize read counts in a direct manner. Indeed, normalization factors are used to normalize total counts. These in turn are used to normalize read counts according to a total count normalization: if N_j is the total number of reads of the sample j and f_j its normalization factor, $N'_j = f_j \times N_j$ is the normalized total number of reads. Then, let $s_j = N'_j / \bar{N}'$ with $\bar{N}'$ the mean of the N'_j s. Finally, the normalized counts of the sample j are defined as $x'_{ij} = x_{ij} / s_j$ where i is the gene index.

	OA384_04112013_SH_OA	356_RNA1_KN_OA	OA385_04112013_SH
TMM	0.94	0.87	

Boxplots are often used to assess the quality of the normalization process, as they show how distributions are globally affected during this process. We expect normalization to stabilize distributions across samples. Figure 8 shows boxplots of raw (left) and normalized (right) data respectively.

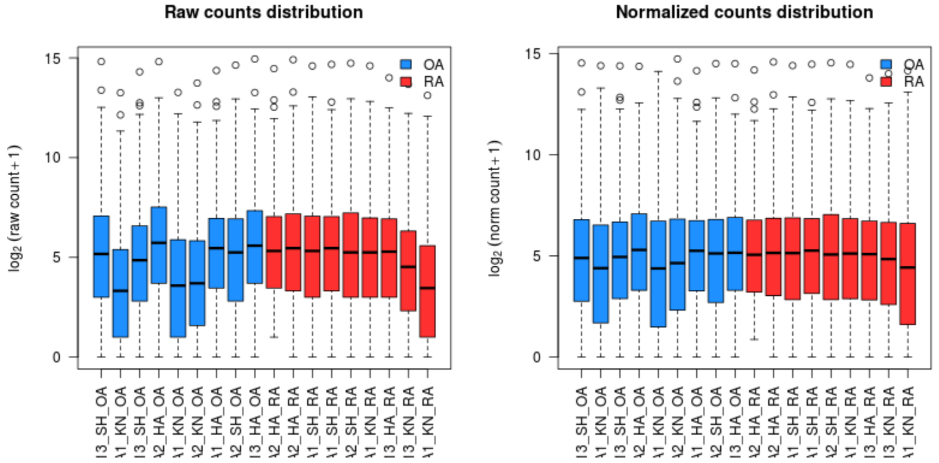

Figure 8: Boxplots of raw (left) and normalized (right) read counts.

6 Differential analysis

6.1 Modelization

edgeR aims at fitting one linear model per feature. For this project, the design used is ~ group and the goal is to estimate the models' coefficients which can be interpreted as $\log_2(\texttt{FC})$. These coefficients will then be tested to get p-values and adjusted p-values.

6.2 Dispersions estimation

The edgeR model assumes that the count data follow a negative binomial distribution which is a robust alternative to the Poisson law when data are over-dispersed (the variance is higher than the mean). The first step of the statistical procedure is to estimate the dispersion of the data.

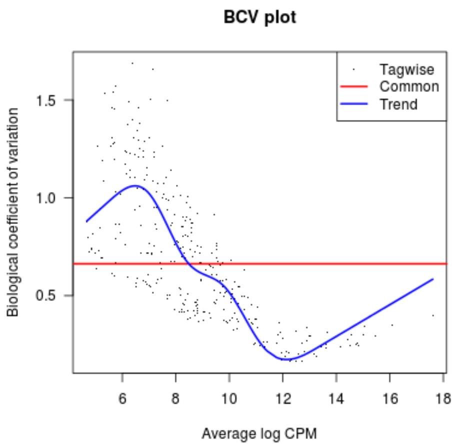

Figure 9: Dispersion estimates.

Figure 9 shows the result of the dispersion estimation step. The x- and y-axes represent the mean count value and the estimated dispersion respectively. Black dots represent empirical dispersion estimates for each feature (from the observed count values). The blue curve shows the relationship between the means of the counts and the dispersions modeled with splines. The red segment represents the common dispersion.

6.3 Statistical test for differential expression

Once the dispersion estimation and the model fitting have been done, edgeR can perform the statistical testing. Figure 10 shows the distributions of raw p-values computed by the statistical test for the comparison(s) done. This distribution is expected to be a mixture of a uniform distribution on $[0, 1]$ and a peak around 0 corresponding to the differentially expressed features.

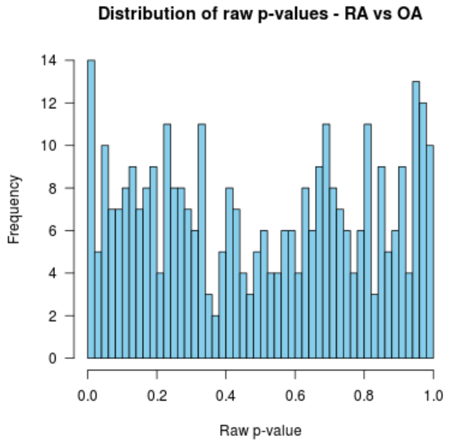

Figure 10: Distribution(s) of raw p-values.

6.4 Final results

A p-value adjustment is performed to take into account multiple testing and control the false positive rate to a chosen level α . For this analysis, a BH p-value adjustment was performed [Benjamini, 1995 and 2001] and the level of controlled false positive rate was set to 0.05.

Figure 11 represents the MA-plot of the data for the comparisons done, where differentially expressed features are highlighted in red. A MA-plot represents the log ratio of differential expression as a function of the mean intensity for each feature. Triangles correspond to features having a too

low/high $\log_2(\text{FC})$ to be displayed on the plot.

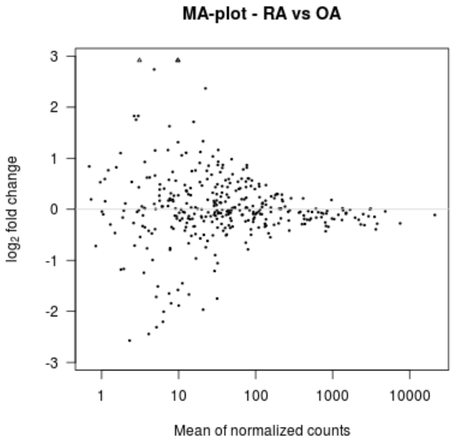

Figure 11: MA-plot(s) of each comparison. Red dots represent significantly differentially expressed features.

Figure 12 shows the volcano plots for the comparisons performed and differentially expressed features are still highlighted in red. A volcano plot represents the log of the adjusted P value as a function of the log ratio of differential expression.

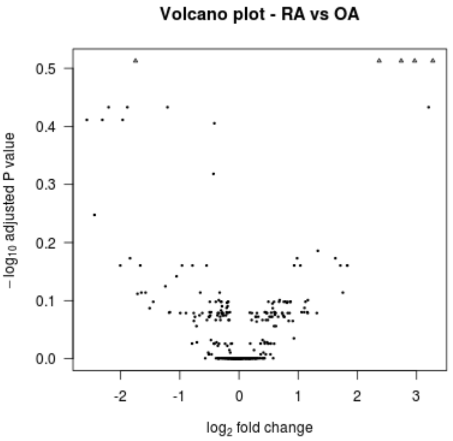

Figure 12: Volcano plot(s) of each comparison. Red dots represent significantly differentially expressed features.

Full results as well as lists of differentially expressed features are provided in the following text files which can be easily read in a spreadsheet. For each comparison:

- TestVsRef.complete.txt contains results for all the features;
- TestVsRef.up.txt contains results for up-regulated features. Features are ordered from the most significant adjusted p-value to the less significant one;
- TestVsRef.down.txt contains results for down-regulated features. Features are ordered from the most significant adjusted p-value to the less significant one.

These files contain the following columns:

- Id: unique feature identifier;
- sampleName: raw counts per sample;
- norm.sampleName: rounded normalized counts per sample;
- baseMean: base mean over all samples;
- OA and RA: means (rounded) of normalized counts of the biological conditions;
- FoldChange: fold change of expression, calculated as $2^{\log_2(\text{FC})}$;
- log2FoldChange: $\log_2(\text{FC})$ as estimated by the GLM model. It reflects the differential expression between Test and Ref and can be interpreted as $\log_2(\frac{\text{Test}}{\text{Ref}})$. If this value is:
  - around 0: the feature expression is similar in both conditions;
  - positive: the feature is up-regulated ($\text{Test} > \text{Ref}$);
  - negative: the feature is down-regulated ($\text{Test} < \text{Ref}$);
- pvalue: raw p-value from the statistical test;
- padj: adjusted p-value on which the cut-off α is applied;
- tagwise.dispersion: dispersion parameter estimated from feature counts (i.e. black dots on figure 9);
- trended.dispersion: dispersion parameter estimated with splines (i.e. blue curve on figure 9).

7 R session information and parameters

The versions of the R software and Bioconductor packages used for this analysis are listed below. It is important to save them if one wants to re-perform the analysis in the same conditions.

R version 3.2.2 (2015-08-14) Platform: x86_64-pc-linux-gnu (64-bit) Running under: Ubuntu 14.04.3 LTS

locale: [1] LC_CTYPE=en_US.UTF-8 LC_NUMERIC=C
[3] LC_TIME=en_US.UTF-8 LC_COLLATE=C
[5] LC_MONETARY=en_US.UTF-8 LC_MESSAGES=en_US.UTF-8
[7] LC_PAPER=en_US.UTF-8 LC_NAME=C
[9] LC_ADDRESS=C LC_TELEPHONE=C
[11] LC_MEASUREMENT=en_US.UTF-8 LC_IDENTIFICATION=C

attached base packages: [1] parallel stats4 methods stats graphics grDevices utils
[8] datasets base

other attached packages: [1] SARTools_1.1.0 xtable_1.7-4
[3] edgeR_3.10.4 limma_3.24.15
[5] DESeq2_1.8.1 RcppArmadillo_0.5.600.2.0 [7] Rcpp_0.12.1 GenomicRanges_1.20.8
[9] GenomeInfoDb_1.4.3 IRanges_2.2.9
[11] S4Vectors_0.6.6 BiocGenerics_0.14.0

loaded via a namespace (and not attached): [1] formatR_1.2.1 RColorBrewer_1.1-2 futile.logger_1.4.1 [4] plyr_1.8.3 XVector_0.8.0 futile.options_1.0.0 [7] tools_3.2.2 rpart_4.1-10 digest_0.6.8
[10] evaluate_0.8 RSQLite_1.0.0 annotate_1.46.1
[13] gtable_0.1.2 lattice_0.20-33 DBI_0.3.1
[16] proto_0.3-10 gridExtra_2.0.0 knitr_1.11
[19] genefilter_1.50.0 stringr_1.0.0 cluster_2.0.3
[22] locfit_1.5-9.1 nnet_7.3-11 grid_3.2.2
[25] Biobase_2.28.0 AnnotationDbi_1.30.1 XML_3.98-1.3
[28] survival_2.38-3 BiocParallel_1.2.22 foreign_0.8-66
[31] latticeExtra_0.6-26 Formula_1.2-1 geneplotter_1.46.0
[34] ggplot2_1.0.1 reshape2_1.4.1 lambda.r_1.1.7
[37] magrittr_1.5 codetools_0.2-14 scales_0.3.0
[40] Hmisc_3.17-0 MASS_7.3-44 splines_3.2.2
[43] colorspace_1.2-6 stringi_0.5-5 acepack_1.3-3.3
[46] munsell_0.4.2

Parameter values used for this analysis are:

- workDir: /home/chazarain/R-L/edgeR
- projectName: stats-edgeR
- author: Your name
- targetFile: ../groups
- rawDir: ..
- featuresToRemove: alignment_not_unique, ambiguous, no_feature, not_aligned, too_low_aQual
- varInt: group
- condRef: OA
- batch: NULL
- alpha: 0.05
- pAdjustMethod: BH
- cpmCutoff: 1
- gene.selection: pairwise
- normalizationMethod: TMM
- colors: dodgerblue, firebrick1, MediumVioletRed, SpringGreen

8 Bibliography

- R Core Team, **R: A Language and Environment for Statistical Computing**, *R Foundation for Statistical Computing*, 2014
- Gentleman, Carey, Bates et al, **Bioconductor: Open software development for computational biology and bioinformatics**, *Genome Biology*, 2004
- Robinson and Smyth, **Moderated statistical tests for assessing differences in tag abundance**, *Bioinformatics*, 2007
- Robinson and Smyth, **Small-sample estimation of negative binomial dispersion, with applications to SAGE data**, *Biostatistics*, 2008
- Robinson, McCarthy and Smyth, **edgeR: a Bioconductor package for differential expression analysis of digital gene expression data**, *Bioinformatics*, 2010
- Dillies, Rau, Aubert et al, **A comprehensive evaluation of normalization methods for Illumina RNA-seq data analysis**, *Briefings in Bioinformatics*, 2012
- Schulze, Kanwar, Golzenleuchter et al, **SERE: Single-parameter quality control and sample comparison for RNA-Seq**, *BMC Genomics*, 2012
- McCarthy, Chen and Smyth, **Differential expression analysis of multifactor RNA-Seq experiments with respect to biological variation**, *Nucleic Acids Research*, 2012
- Wu, Wang and Wu, **A new shrinkage estimator for dispersion improves differential expression detection in RNA-seq data**, *Biostatistics*, 2013
- Benjamini and Hochberg, **Controlling the False Discovery Rate : A Practical and Powerful Approach to Multiple Testing**, *Journal of the Royal Statistical Society*, 1995
- Benjamini and Yekutieli, **The control of the false discovery rate in multiple testing under dependency**, *The Annals of Statistics*, 2001
